# Bacterial Proteasome Activator Bpa (Rv3780) Is a Novel Ring-Shaped Interactor of the Mycobacterial Proteasome

**DOI:** 10.1371/journal.pone.0114348

**Published:** 2014-12-03

**Authors:** Cyrille L. Delley, Juerg Laederach, Michal Ziemski, Marcel Bolten, Daniel Boehringer, Eilika Weber-Ban

**Affiliations:** ETH Zurich, Institute for Molecular Biology & Biophysics, Zurich, Switzerland; University of Padova, Medical School, Italy

## Abstract

The occurrence of the proteasome in bacteria is limited to the phylum of actinobacteria, where it is maintained in parallel to the usual bacterial compartmentalizing proteases. The role it plays in these organisms is still not fully understood, but in the human pathogen *Mycobacterium tuberculosis* (Mtb) the proteasome supports persistence in the host. In complex with the ring-shaped ATPase Mpa (called ARC in other actinobacteria), the proteasome can degrade proteins that have been post-translationally modified with the prokaryotic ubiquitin-like protein Pup. Unlike for the eukaryotic proteasome core particle, no other bacterial proteasome interactors have been identified to date. Here we describe and characterize a novel bacterial proteasome activator of *Mycobacterium tuberculosis* we termed Bpa (Rv3780), using a combination of biochemical and biophysical methods. Bpa features a canonical C-terminal proteasome interaction motif referred to as the HbYX motif, and its orthologs are only found in those actinobacteria encoding the proteasomal subunits. Bpa can inhibit degradation of Pup-tagged substrates *in vitro* by competing with Mpa for association with the proteasome. Using negative-stain electron microscopy, we show that Bpa forms a ring-shaped homooligomer that can bind coaxially to the face of the proteasome cylinder. Interestingly, Bpa can stimulate the proteasomal degradation of the model substrate β-casein, which suggests it could play a role in the removal of non-native or damaged proteins.

## Introduction

Large protease complexes that isolate their active sites on the inner walls of a cylindrical compartment exist in all organisms [1], [2]. The most advanced of these particles is the eukaryotic 20S proteasome, a complex comprised of four rings stacked in αββα order [3], [4]. The central β-rings carry the proteolytic activity and the outer α-rings are responsible for interaction with regulatory particles that are themselves ring-shaped. Actinobacteria are the only bacterial phylum that also features a proteasome in addition to standard bacterial degradation cylinders [5], [6]. Although not essential during normal growth, the bacterial proteasome provides an advantage under stress conditions as encountered by *Mycobacterium tuberculosis* (Mtb) inside host macrophages or more generally during starvation [7]–[10].

For eukaryotes, two types of interaction partners have been described for the proteasome core cylinder (referred to as 20S proteasome), the ATPase-active 19S regulatory complex and the non-ATPase 11S complex (also PA28 or REG) and PA200 [11]–[15]. Both interactors dock to the 20S proteasome via a C-terminal hydrophobic-tyrosine-X motif (HbYX motif) that inserts into hydrophobic pockets on the α-rings of the proteasome [16]. This docking event was shown to open the “proteasomal gate”, an obstruction consisting of the α-subunit N-terminal tails that close off the 20S core particle pores [16]. The 19S regulatory particle recognizes poly-ubiquitinated protein substrates, unfolds them in an ATPase-dependent manner and translocates them into the proteolytic 20S cylinder, where they are processively degraded into short peptides [17]–[20]. A functionally analogous substrate recruitment and degradation system has been described in mycobacteria [21], [22], where the homohexameric proteasomal ATPase ring Mpa (referred to as ARC in other actinobacteria) recognizes protein substrates modified with the prokaryotic ubiquitin-like protein Pup, unfolds them and transfers them into the 20S proteasome for degradation [23], [24]. Mpa also employs a HbYX motif (QYL in Mtb) at its C-terminus for the interaction with the 20S core particle [24]. Curiously, however, despite the presence of this motif, efficient interaction between Mpa and the 20S *in vitro* particle can only be achieved when an open-gate variant of the proteasome is used, in which the N-termini of the α-subunits are truncated [25]. This raises questions about the *in vivo* distribution of proteasomal complexes formed with either Mpa or potential other interactors. Furthermore, it leaves open the possibility that a proteasomal assembly chaperone is needed for promoting a conformational change in the 20S particle supporting gate-opening *in vivo*. Such assembly factors have been described for both eukaryotic as well as archaeal proteasomes and were shown to function in a HbYX motif-dependent fashion [26].

In this study we identify an ATP-independent bacterial proteasome interactor we named Bpa (Rv3780). Although, Bpa shows no sequence homology to the eukaryotic 11S complex, it features the canonical HbYX motif and is conserved in all proteasome harboring actinobacteria. We demonstrate the physical interaction of Bpa with the proteasome using both biochemical and structural methods. Furthermore, we report the ability of Bpa to stimulate proteasomal degradation of β-casein as a model substrate of unfolded proteins.

## Materials and Methods

### Alignment and co-occurrence

The Mtb genome was searched for proteins featuring a C-terminal HbYX motif using a custom Python script. A subset of actinobacterial genomes in accordance with our previous study [27] was chosen for homology and shared synteny searches via BLAST [28] and SyntTax [29], respectively. Co-occurrence of Bpa and PrcAB (the proteasome 20S core) was verified by String search [30].

A multiple sequence alignment of Rv3780 (Bpa) homologs was carried out using ClustalW2 [31] and visualized using Jalview [32]. For genomic placement and orientation of the *pup* locus and *bpa* the NCBI genome data was used.

### Cloning, expression and protein purification

Rv3780 (Bpa) was amplified from genomic DNA of Mtb H37Rv by PCR with Phusion DNA polymerase (New England Biolabs) and ligated into a modified pET24b expression plasmid described previously [33]. BpaΔHbYX was generated by site-directed mutagenesis. Constructs were transformed into *E. coli* BL21(DE3) cells for heterologous expression and induced with 1 mM IPTG after the cultures reached an OD_600_ of 0.8. Cells were cracked by sonication and proteins were purified by Ni-NTA affinity chromatography followed by a reverse Ni-NTA step after Tobacco Etch Virus (TEV) endopeptidase cleavage of the His-tag. Finally, the sample was applied to a Superdex 200 size exclusion chromatography column and run in buffer S (50 mM HEPES-NaOH pH 7.5 (4°C), 150 mM NaCl, 10% (v/v) Glycerol and 1 mM EDTA).

β-casein was purified from bovine milk casein mix (Sigma) using anion exchange chromatography.

To test for protein interaction using a bacterial adenylate cyclase two-hybrid system (BACTH, see next paragraph), the target proteins Msmeg_6365 (^Msm^Bpa) corresponding to Rv3780 in Mtb and Msmeg_3894 (^Msm^PrcA) were fused to the C-terminus of the catalytic adenylate cyclase domains T25 and T18, respectively. Both genes were amplified from *M. smegmatis* MC^2^-155 genomic DNA using Phusion DNA polymerase (New England Biolabs) and cloned into pKT25 and pUT18C vectors using the Gibson Assembly (New England Biolabs) approach. The open-gate variant of ^Msm^PrcA (proteasome α-subunit) and a mutated variant of ^Msm^Bpa lacking the C-terminal HbYX motif, ^Msm^BpaΔHbYX, were generated by site-directed mutagenesis. The T18-*zip* and T25-*zip* gene fusions, which served as positive controls, were obtained from Euromedex.

### Protein interaction study using BACTH

To assess the interaction between ^Msm^PrcA and ^Msm^Bpa, a BACTH (bacterial adenylate cyclase two-hybrid) assay was performed as described by Karimova *et al.* [34]. Briefly, *E. coli* BTH101 *cya* ^−^ cells were co-transformed with the pKT25 and pUT18C fusion constructs in different combinations. The co-transformants were selected on LB agar plates supplemented with 100 µg/ml ampicillin and 50 µg/ml kanamycin by incubation for 48 h at 30°C. Several clones were then grown overnight at 30°C in liquid LB medium containing both antibiotics and 0.5 mM IPTG. Subsequently, 40 µl of the bacterial culture were transferred onto MacConkey agar containing the same antibiotics with addition of 1% maltose and incubated for 24 h at 30°C. As a negative control bacteria were co-transformed with empty pKT25 and pUT18C vectors and as a positive control the cells were co-transformed with pKT25-*zip* and pUT18-*zip* plasmids. For quantitative analysis, a β-galactosidase assay was performed in triplicates as described previously by Battesti and Bouveret [35] and Miller units were calculated according to Miller [36].

### Proteasome competition assay

*In vitro* pupylated PanB (PanB-Pup) was generated as described previously [37]. Prior to the degradation time course, PanB-Pup (2 µM protomer) was incubated with Δ7PrcAB (0.1 µM 28 mer), 5 mM ATP, 40 mM phosphocreatine, 0.4 U/ml creatine phosphokinase, 1 mM DTT in buffer R (50 mM Tris-HCl pH 7.5, 150 mM NaCl, 20 mM MgCl_2_, 10% (v/v) Glycerol) for 30 s in the presence or absence of Bpa or BpaΔHbYX (14 µM protomer). The degradation reaction was started by addition of 0.2 µM Mpa (hexamer). The reaction was sampled at the indicated time points by quenching into Laemmli buffer and analyzed by SDS-PAGE stained with Coomassie Brilliant Blue.

### Size Exclusion Chromatography

Size exclusion chromatography was performed on a Superdex 200 (10/300) GL column (GE Healthcare) with an ÄKTA Purifier system. All runs were performed at room temperature and a flow rate of 1 ml/min in buffer R. Bpa was injected at a concentration of 64 µM (protomer) in a volume of 100 µl and was detected by absorption at 230 nm. Molecular weight standards were run under the same conditions and absorption was measured at 280 nm. A standard curve with the formula: Kav = −0.133*ln(MW) +1.869 was obtained to calculate the theoretical molecular weight for Bpa.

### Pull-downs

Strep-tagged PrcAB variants were incubated at a concentration of 2 µM (holoparticle) or 4 µM (half-proteasome particles) with 24 µM Bpa or BpaΔHbYX (protomer) in buffer R for two hours at room temperature in a volume of 50 µl. The reaction mixture was further incubated on 25 µl equilibrated Strep-Tactin Sepharose (IBA) in spin columns at room temperature for one hour with gentle shaking. Flowthrough (50 µl), wash (5×50 µl), and elution fractions (3×50 µl, containg 2.5 mM desthiobiotin) were collected by centrifugation at 400 rcf for 30 s in a table-top microcentrifuge. All fractions were supplemented with Laemmli buffer and submitted to SDS-PAGE, then stained with Coomassie Brilliant Blue.

### Proteasome maturation

Recombinantly expressed half-proteasomes (full length PrcA and PrcB-Strep) were purified as described [24]. Maturation was performed at 37°C on a table-top thermo shaker in 70 µl volume containing 0.4 µM half proteasomes and 24 µM Bpa/BpaΔHbYX (protomer). Samples were taken at the indicated time-points and run on SDS-PAGE, then stained with Coomassie Brilliant Blue.

### Negative Stain EM

Electron Microscopy specimens were prepared by applying a drop of Bpa protein solution at a concentration of 2.8 µM or, for complex formation, at a concentration of 600 nM together with 40 nM open-gate PrcAB (Δ7PrcAB) in buffer R on freshly glow-discharged, carbon-coated 300 mesh copper grids (Quantifoil) for 30 s. The sample drop was blotted away and the grid was stained with 1% (w/v) aqueous uranyl acetate for 30 s. The samples were imaged in a FEI Morgagni 268 transmission electron microscope operating at 100 kV with a nominal magnification of 60′000.

### Peptidase activity

10 nM PrcAB were incubated with 6 µM Bpa (protomer) and 150 µM L-Succcinyl-Leu-Leu-Val-Tyr-4-Amino-7-Methylcoumarin (L-Suc-LLVY-AMC) in buffer R with 1.5% DMSO. Peptidase activity was measured by fluorescence detection of released AMC molecules with an excitation wavelength of 360 nm and emission set at 460 nm. The experiments were performed in 50 µl reaction volume in Corning non-binding 96-well half area assay plates in a BioTek Synergy 2 plate reader (Tungsten light source, top 50% optics position, sensitivity: 60).

### Casein Degradation

β-casein (27 µM) was incubated at 37°C together with 6 µM Bpa or BpaΔHbYX (protomer), 0.2 µM PrcAB or Δ7PrcAB, respectively, 1 mM ATP in buffer R supplemented with 1 mM DTT. The reaction was started with the addition of proteasome and sampled at the indicated time points. A control reaction without proteasome was performed. The samples were quenched in Laemmli buffer and visualized on Coomassie stained SDS-PAGE.

## Results

### Mtb genome search for proteins with a C-terminal HbYX proteasome interaction motif

It has been shown that the mycobacterial proteasomal ATPase Mpa employs a C-terminal hydrophobic-tyrosine-X motif (HbYX motif) in the interaction with the 20S proteasome (also referred to as PrcAB in this study) [23], [24]. So far, Mpa was the only identified interactor of the mycobacterial proteasome [24], [25]. In an attempt to identify possible alternative proteasomal regulators, we searched the genome of Mtb for genes encoding proteins that feature a C-terminal HbYX motif. For the hydrophobic residue at position −3 we excluded aspartate, glutamate, lysine, arginine, serine and cysteine. Furthermore, we limited the search to those Mtb genes for which the HbYX motif was conserved also in the homologous genes of other proteasome-bearing actinobacteria. We also required the search to return only those genes that showed co-occurrence with the proteasomal subunit genes *prcA* and *prcB*. They should not feature homologs in either corynebacteria or bifidobacteria, but on the other hand should also not be limited to only the mycobacterial domain.

**Figure 1:**
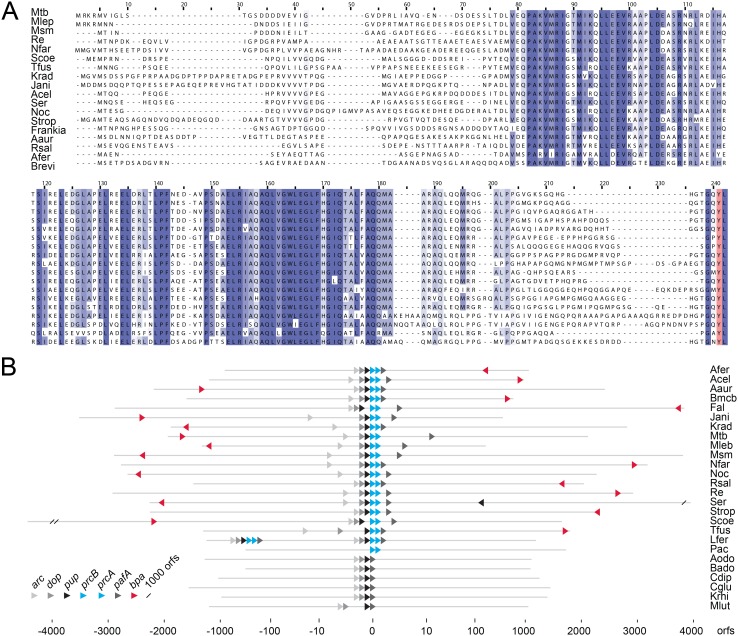
Bpa is conserved in actinobacteria encoding the proteasomal core particle genes. A, Multiple sequence alignment of Bpa orthologs (Rv3780 in Mtb) from different actinobacteria. The predominant residues in positions with an identity score above 0.5 are shaded in blue where increasing similarity is indicated by a gradient from light to dark blue. The completely conserved penultimate tyrosine of the HbYX motif is colored in red. B, Occurrence and location of the Pup-proteasome gene locus and the bpa gene. Each line represents the location of all open reading frames (orfs) of a bacterium in relation to the Pup proteasome gene locus. The position and orientation (if a corresponding homolog exists) of the proteasomal genes *prcB* and *prcA* are indicated by turquoise and *bpa* by a red arrow, respectively. The pupylation genes are given in shades of grey. Organisms are abbreviated as follows: *M. tuberculosis* (Mtb), *M. leprae* (Mlep), *M. smegmatis* (Msm), *N. farcinica* (Nfar), *R. erythropolis* (Re*), S. coelicolor* (Scoe), *T. fusca* (Tfus), *K. radiotolerans* (Krad), *Janibacter sp.* (Jani), *A. cellulolyticus* (Acel), *S. erythraea* (Ser), *Nocardioides sp.* (Noc), *S. tropica* (Strop), *Frankia sp.* (Frankia), *A. aurescens* (Aaur), *R. salmoninarum* (Rsal), *A. ferrooxidans* (Afer), *Brevibacterium sp.* (Brevi), *B. mcbrellneri* (Bmcb), *F. alni* (Fal), *L. ferrooxidans* (Lfer), *P. acnes* (Pac), *A. odontolyticus* (Aodo), *B. adolescentis* (Bado), *C. diphtheria* (Cdip), *C. glutamicum* (Cglu), *K. rhizophila* (Krhi), *M. luteus* (Mlut).

Our search returned four genes that fulfilled these requirements, two of which coded for proteins of unknown function. Interestingly, one of these proteins, Rv3780 (in this study referred to as Bpa), carries at its C-terminus the same four residues as the proteasomal ATPase Mpa, which means both feature the GQYL-motif that is in Mpa responsible for interaction with the proteasome. A multiple sequence alignment of Bpa with its orthologs from 16 actinobacteria using ClustalW2 shows, that, aside from a region of 30–40 residues at the N-terminus and a stretch of 15–20 residues close to the C-terminus, the protein sequences exhibit high homology. The four C-terminal residues are again highly conserved, not only the penultimate tyrosine but the entire GQYL motif (Figure 1A). The *bpa* gene occurs only in those actinobacteria that also carry the proteasomal subunit genes (*prcA* and *prcB*) and is absent in those that do not (Figure 1B). *Propionibacterium acnes* is an exception, as it harbors the proteasomal subunit genes, but neither features the proteasomal ATPase ARC nor a gene for Bpa. The fact that *Leptospirillum ferrooxidans* harbors the Pup-proteasome-system but not Bpa, only stresses that this bacterium, which is not a member of the actinobacteria, has obtained the Pup-proteasome locus by horizontal gene transfer as suggested previously [1], [27]. The conserved C-terminal HbYX motif along with the co-occurrence of Bpa with the proteasomal subunit genes (Figure 1B) suggests a role for Bpa as a potential proteasomal interaction partner. However, there is no clear spacial relation to the proteasome gene locus. In fact, Bpa and its orthologs are encoded at loci far away from the Pup-proteasome gene locus, with a gene neighborhood that appears unconnected to proteasomal function.

### Bpa associates with the proteasome in a HbYX motif dependent manner

**Figure 2:**
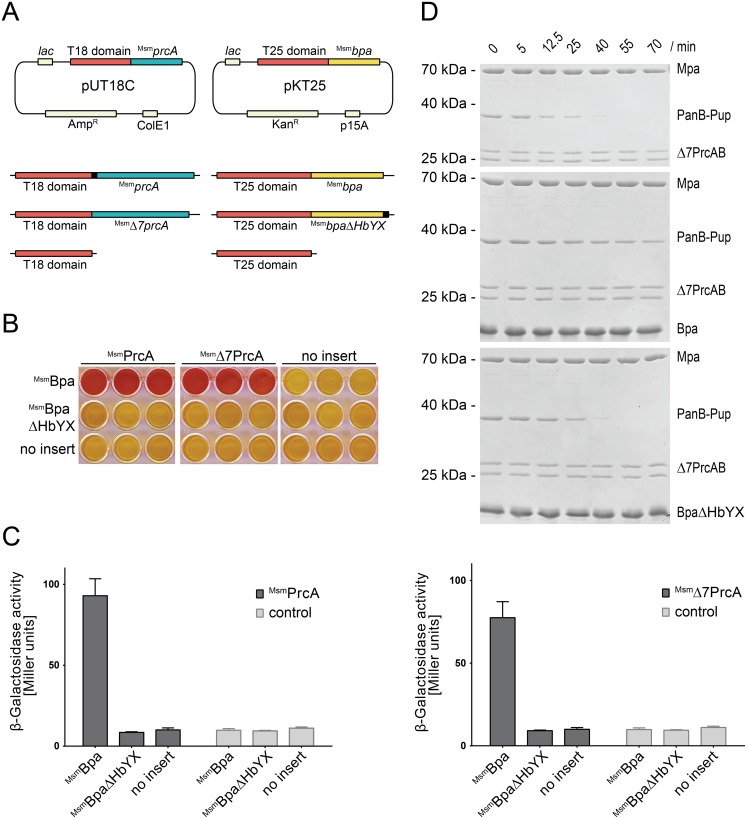
Bpa interacts with the 20S proteasome through its C-terminal HbYX motif. A, Vector constructs for the bacterial two-hybrid screen. PrcA and Δ7PrcA were fused to the C-terminus of the catalytic T18 domain, Bpa and BpaΔHbYX to the C-terminus of the catalytic T25 domain of adenylate cyclase. B, MacConkey agar matrix of all pairwise combinations of the pUT18C and pKT25 constructs in triplicates. Successful interaction between T18 and T25 switches the colony from lac− to lac+ and the resulting acidification of the agar is visualized by the pH indicator turning red. C, A quantitative β-galactosidase assay of the same hybrid experiments as shown in B. The assay was performed on chloroform-treated *E. coli* cells grown overnight in liquid LB medium containing 0.5 mM IPTG. The background activity is indicated by the negative control (no insert, corresponding to pKT25 and pUT18C carrying only the adenylate cyclase domains without fusion). Bars represent averages ± SEM of at least three replicates. D, Mpa-mediated proteasomal PanB-Pup degradation is inhibited in presence of association-competent Bpa but not in presence of BpaΔHbYX. Concentrations: Mpa (0.2 µM), 20S proteasome (0.1 µM), Bpa or BpaΔHbYX (14 µM protomer).

To test our hypothesis that Bpa might physically interact with the 20S proteasome particle or individual proteasomal subunits, we carried out a bacterial adenylate cyclase-based two-hybrid analysis, constructed around the reconstitution of adenylate cyclase activity in an *E. coli* strain lacking the gene encoding this enzyme. Adenylate cyclase deficient *E. coli* cells were co-transformed with a plasmid expressing the Bpa homolog of *Mycobacterium smegmatis* (^Msm^Bpa) tethered to the C-terminus of the adenylate cyclase T25 domain and another plasmid carrying the proteasomal α-subunit gene (*^Msm^prcA*) tethered to the C-terminus of the adenylate cyclase T18 domain (Figure 2A). As cyclic AMP (cAMP) produced by reconstituted adenylate cyclase stimulates transcription of the *lac* operon, a β-galactosidase assay was employed in liquid culture to test for formation of an active catalytic adenylate cyclase domain in case of physical interaction taking place between Bpa and the proteasomal subunit (Figure 2C). In addition, presence of interaction was tested on MacConkey agar (Figure 2B), which turns red upon interaction of the two adenylate cyclase subdomains. As a control, and to test if the interaction is dependent on the HbYX motif, we included a setup where the C-terminal HbYX motif of ^Msm^Bpa was removed (^Msm^BpaΔHbYX). A positive interaction signal, indicated by the red color of the MacConkey agar wells, was obtained with cells expressing ^Msm^Bpa and the proteasomal α-subunit (either full-length or lacking the first seven residues) tethered to the two adenylate cyclase domains (Figure 2B, top row, first and second triplet of wells). No color development was observed when the ^Msm^BpaΔHbYX variant was used (second row), indicating that the interaction is dependent on the HbYX motif.

We then produced Bpa from Mtb (Rv3780) recombinantly to characterize the interaction *in vitro*. As we predict Bpa to interact with the proteasome in a manner analogous to the proteasomal ATPase Mpa based on the common HbYX motif, we carried out a competition assay, where Mpa-proteasome mediated degradation of a pupylated substrate was performed in absence and in presence of an excess of either wild type Bpa or a variant lacking the four C-terminal residues (BpaΔHbYX). While in absence of Bpa the open-gate proteasome in complex with Mpa degrades the pupylated substrate (PanB-Pup) fully within about 40 min under the chosen conditions, the degradation is strongly inhibited in presence of an excess of Bpa (Figure 2D). This competitive inhibition is dependent on the HbYX motif, as the truncated variant BpaΔHbYX does not prevent degradation of the pupylated substrate within the same time frame (Figure 2D).

### Bpa forms homooligomeric ring assemblies and stacks to the ends of the proteasomal cylinder

**Figure 3:**
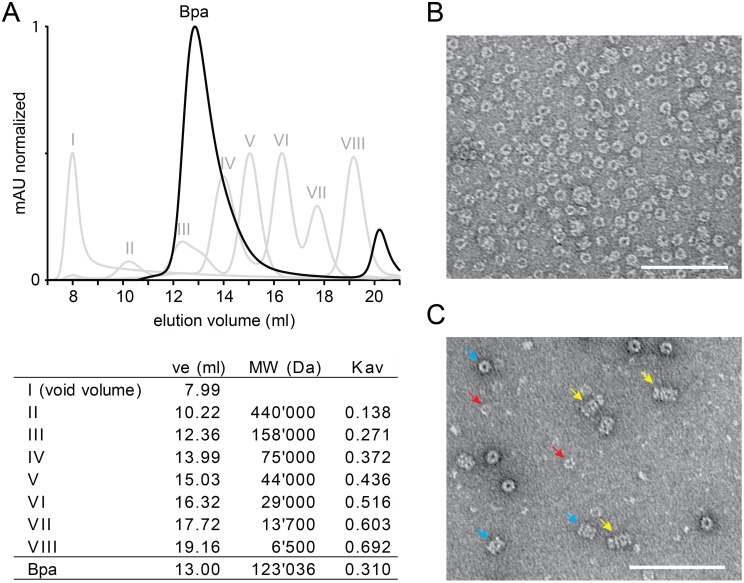
Bpa assembles into ring-shaped homo-oligomeric complexes and associates coaxially with the proteasome. A, Analytical gel filtration profile of Bpa on a Superdex 200 column (black curve). Molecular size standards (I–VIII, grey curves) were run under the same conditions. The calculated molecular weight of Bpa at 13 ml elution volume is 123 kDa assuming an overall globular shape of the oligomer. B, Electron micrograph of negatively stained Bpa showing top views of the ring-shaped Bpa complexes at a magnification of 60′000. C, Electron micrograph of negatively stained Bpa and Δ7PrcAB shows top views of Bpa (examples indicated by red arrows) and Δ7PrcAB (turquoise arrows) as well as side views of the coaxially stacked complexes formed between Bpa and Δ7PrcAB (marked with yellow arrows). The scale bars in B and C both indicate 100 nm.

To determine the assembly state of Bpa, size exclusion chromatography was performed. From a Superdex 200 gel filtration column, Bpa elutes as a single peak at a position roughly equivalent to a complex of 123 kDa based on a comparison with the molecular size standards, however tailing out slightly toward the lower molecular weight end (Figure 3A). As the protomer has a mass of 19.7 kDa (Figure S1A), this suggests a homo-oligomeric assembly of six subunits that, based on the slight tailing, might exhibit either a tendency to fall apart or show a mix of assembly states or conformations. As the shape of the oligomer is likely not globular, a heptameric assembly, as observed for the eukaryotic proteasome activators, cannot be excluded. We can rule out the possibility that other impurities contribute to the shape of the gel filtration peak since the Bpa protein preparation is more than 95% pure (Figure S1B).

To further assess the assembly state as well as to investigate the shape of the particle, negative stain electron micrographs of the recombinantly produced Bpa protein were recorded. The micrographs show particles with a ring-like architecture. The rings display as top views with an overall diameter of roughly 120 Å and a hole in the center (Figure 3B), indicated by accumulating stain.

**Figure 4:**
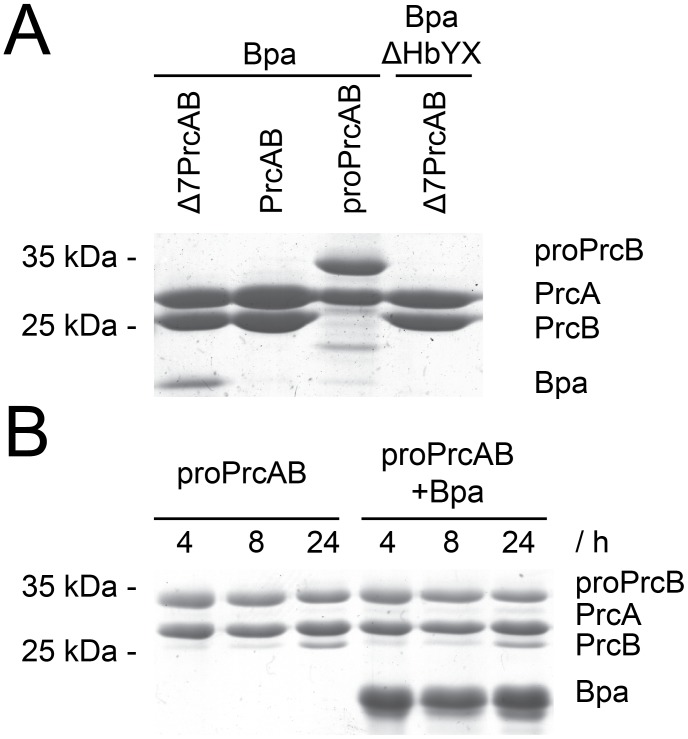
Bpa can be retained by immobilized Δ7PrcAB or half-proteasomes. A, Elution fractions of the pull-downs of Bpa or BpaΔHbYX with different Strep-immobilized proteasome particles. Strep-tagged proteasome particles were eluted from Strep-Tactin Sepharose with 2.5 mM desthiobiotin and the elution fractions were visualized on Coomassie stained SDS-PAGE. Bpa binds to Δ7PrcAB, while BpaΔHbYX does not. PrcAB interaction with Bpa is not detectable, while half-proteasomes (proPrcAB) retain a small amount of Bpa. B, Maturation of half-proteasomes at 37°C for 24 hours followed by SDS-PAGE. No difference in processing speed can be observed in the reactions supplemented with Bpa compared to PrcAB alone within this time frame.

The ring-shaped assembly mode of Bpa suggests that it interacts with the proteasomal 20S core through a ring-stacking association to the cylinder ends. In an attempt to observe this interaction, negatively stained EM micrographs were recorded in the presence of the 20S proteasome. To increase the chance for observing the complex on the grid, pull-down experiments were performed with different forms of the proteasome core, to see which might give the most stable interaction. The pull-down analysis indicated that open-gate proteasome interacts more stably with Bpa than the wild type proteasome, which did not yield a detectable band in Coomassie-stained SDS-PAGE (Figure 4A). Immature proteasome particles (proPrcAB) were in this context also tested for interaction with Bpa to account for a potential role in proteasome assembly. The pull-down analysis using half-proteasomes resulted in a barely detectable band for Bpa, indicating less interaction with half-proteasomes than with open-gate mature proteasomes but a somewhat stronger interaction than with the mature wild-type proteasome particle under the same conditions. Nevertheless, processing of the half-proteasomes was not stimulated by the presence of Bpa (Figure 4B).

EM micrographs were therefore recorded in the presence of open-gate 20S proteasome particles (Δ7PrcAB). Figure 3C shows a section of a grid with top and side views of the open-gate 20S particle (turquoise arrows), top views of Bpa (red arrows) as well as a few side views of the Bpa-proteasome complex (yellow arrows). All Bpa-proteasome complexes are capped on one side by the Bpa ring, adding an additional striation to the four-layered side view of the proteasome particle. Although we have no easy way of identifying top views of the complex, the pores of the Bpa and proteasomal rings are likely aligned, since the rings in side view appear to be coaxial.

### Bpa acts as a proteasomal activator

**Figure 5:**
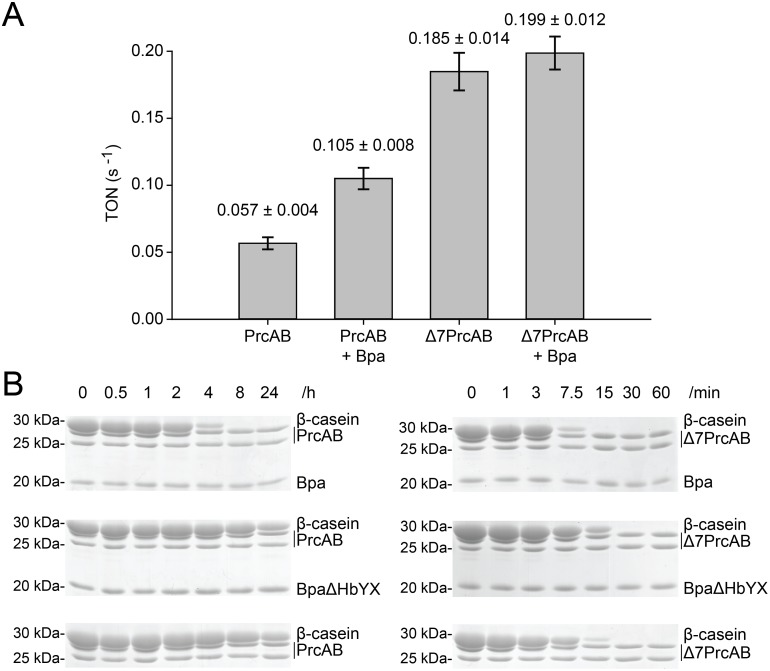
Bpa acts as a proteasomal activator. A, Peptidase activity measured by fluorescence detection of AMC molecules (λ_ex_: 360 nm, λ_em_: 460 nm) released from the fluorogenic peptide L-Suc-LLVY-AMC (150 µM). PrcAB exhibits a turnover number (TON) of 0.057±0.004 s^−1^, which is more than three times less active than Δ7PrcAB with a TON of 0.185±0.014 s^−1^. Addition of an excess of Bpa to the reaction increases peptidase activity of PrcAB about 1.8 fold to a TON of 0.105±0.008 s^−1^. B, Degradation of β-casein (27 µM) at 37°C by Δ7PrcAB or PrcAB (0.2 µM) in presence or absence of Bpa or BpaΔHbYX (6 µM protomer) is sampled at different time points and analyzed by Coomassie-stained SDS-PAGE.

The inhibition of Mpa-driven degradation of a pupylated substrate indicates that Bpa competes with Mpa for binding to the 20S proteasome α-rings, and the micrographs detected complexes with the Bpa ring aligned coaxially with the proteasome potentially allowing passage of substrates. To test whether association of Bpa with the 20S proteasome can stimulate the proteasome active sites we carried out peptidase assays in presence and absence of the novel interactor. The fluorogenic model peptide L-Succinyl-LLVY-AMC was used at a concentration of 150 µM to measure peptide degradation rates (Figure 5A). In presence of Bpa the peptidase activity is stimulated by roughly two-fold, suggesting that Bpa influences the conformation of the 20S complex upon interaction.

As a non-ATPase proteasome interactor, Bpa cannot provide the necessary energy to unfold native proteins for degradation. However, unfolded polypeptides could serve as potential substrates for the Bpa-PrcAB complex. We therefore also tested for degradation of the model protein casein, which is known to expose hydrophobic moieties and to adopt a non-globular, extended structure mimicking unfolded proteins. Casein degradation was measured in absence and presence of Bpa with both wild type proteasome complex as well as open-gate proteasome (Figure 5B). In absence of the proteasome activator, wild type proteasome particles are unable to degrade casein within the time frame of 8 hours. Even after 24 hours only a portion of the casein has been degraded by the wild type proteasome alone (Figure 5B, left panel). When Bpa is present, however, casein is degraded fully within the 8 hour time frame, clearly demonstrating that Bpa is capable of stimulating degradation of a protein substrate in extended conformation. The open-gate variant of the proteasome is able to degrade casein in absence of the activator (Figure 5B, right panel). But even here, addition of the activator further stimulates this activity.

## Discussion

For eukaryotic proteasomes, several activators have been described that share the principle ring-shaped architecture and the ability to stack to the distal ends of the proteasomal cylinder, thereby regulating or modulating access and entry of protein substrates into the proteolytic chamber [14], [15]. The activators fall into two categories, energy-dependent ATPase complexes and energy-independent non-ATPase rings. The eukaryotic 19S regulatory particle with its hexameric ATPase ring associates with the proteasome to form the 26S proteasome, the main extralysosomal route for protein degradation, responsible for the degradation of substrates via the ubiquitin-proteasome pathway [4], [38], [39]. In the proteasome-harboring mycobacteria and other actinobacteria, the analogous complex is formed between the proteasomal ATPase Mpa (ARC in other actinobacteria) and carries out the degradation of Pup-tagged substrates [40], [41]. However, unlike the eukaryotic 26S complex, the Mpa-proteasome is not a house-keeping degradation complex, but appears to play a role only under specific environmental pressures [7], [10]. The eukaryotic proteasome is known to also form alternate complexes with non-ATPase activators like for example the various forms of the 11S regulator (also called PA28 or PA26 in some organisms) and the PA200 (Blm10 in yeast) [42], [43]. For the actinobacterial proteasome, no other interactor besides Mpa/ARC had been described so far.

With the identification of the bacterial proteasome activator Bpa in this study, it is becoming apparent that the actinobacterial system shares the modular nature of the eukaryotic counterpart, and that alternative complexes can be assembled. Eukaryotic energy-independent activators were demonstrated to form predominantly heptameric rings. Our electron microscopic images are in agreement with either a hexameric or heptameric state, and high resolution structural analysis will be required to conclusively determine the number of subunits in the ring. Quantitative mass spectrometric analysis of the Mtb proteome, which provides estimates on the absolute abundance for 55% of all Mtb proteins under standard culture conditions [44], shows that the proteasome particle is present at about 3-fold excess over Mpa hexamers and Bpa is detected at levels equivalent to a roughly 10-fold excess over Mpa hexamers (assuming a hexameric or heptameric ring assembly state for Bpa). This suggests that different proteasome complexes might exist in parallel under certain conditions. However, it would be expected that further regulatory mechanisms induce increased production of one or the other proteasome activator in response to different environmental conditions.

Our study presents several lines of evidence demonstrating interaction of Bpa with the α-rings of the proteasome complex. Interestingly, interaction can be detected with various forms of the proteasome complex. For instance, the bacterial two-hybrid analysis was carried out with the proteasomal α-subunit in absence of the β-subunit. This not only shows that interaction occurs at the α-rings of the proteasome particle without involvement of the β-rings, but also suggests that Bpa can, in principle, interact with α-rings alone. It should be mentioned that the proteasome complex of another actinobacterium, *Rhodococcus erythropolis*, was shown to assemble through an αβ-dimer intermediate that oligomerizes further to form half-proteasomes, precluding single α-rings [45]. Although the α-subunits alone are reportedly not able to form rings on their own, it is possible that an equilibrium exists between the predominantly monomeric form and a small population of rings that the Bpa could bind. Alternatively, unassembled α-subunits might bind to the Bpa ring. This latter possibility is less likely based on how eukaryotic proteasome interactors bearing the HbYX motif interact with the 20S particle. There, structural analyses have revealed that the C-terminal HbYX motif docks into pockets at the interfaces between two α-subunit protomers in the α-ring [42], [43], [46].

Throughout the study two versions of the proteasome particle were used, the wild-type form, featuring full-length α-N-termini that presumably close off the proteasome ends, and a so-called “open-gate” variant, where seven residues of the N-terminus were deleted. This variant is commonly used for *in* ****** *vitro* studies of the Mpa-proteasome complex, as the wild-type proteasome forms only transient interactions with the Mpa ring [24], [25]. Our *in* ****** *vitro* experiments with Bpa indicate that it also only forms stable complexes, detectable by negative stain electron microscopy, with the open-gate proteasome variant. Nevertheless, it clearly interacts productively with the wild-type proteasome as demonstrated by the stimulated degradation of β-casein in presence of Bpa.

Remarkably, in a pull-down assay on purified recombinantly produced Bpa using immobilized Strep-tagged proteasome particles, a very faint Bpa-band is detected in the coelution with half-proteasomes. A possible role in the proteasome assembly pathway can thus not be completely excluded. However, an acceleration of maturation from the unprocessed β-form to the processed form is not observed in presence of Bpa.

The shared C-terminal interaction motif GQYL suggests a similar mode of interaction between Bpa or Mpa and the proteasome, despite the fact that the two proteins do not exhibit sequence homology beyond that. The curiously transient interaction with the wild-type proteasome for both interactors and stabilization of the interaction with truncation of the α-N-termini also appears to be a shared property. This leaves open the question whether interaction *in* ****** *vivo* can possibly be as transient or whether additional regulatory factors might be involved that stabilize complex formation, perhaps even selectively for one or the other activator. These factors could either come in the form of additional macromolecular binding partners or, alternatively, post-translational modifications, such as phosphorylation, could play a role in modulating the affinity. Notably, a very recent study shows that the α-subunit of the Mtb proteasome is phosphorylated at three threonine residues and that this leads to enhanced degradation of a known proteasomal substrate *in* ****** *vivo* [47].

The observation that Bpa stimulates the degradation of β-casein by the wild type proteasome suggests that this alternate degradation complex plays a role in the removal of poorly structured proteins. The occurrence of such protein conformations could be increased under the influence of various stresses encountered by the bacteria. In case of Mtb this could be the hostile environment of the host macrophages where nitrosative stress and higher levels of reactive oxygen species contribute to protein damage. *In* ****** *vivo* studies under a range of stress conditions or in infection models will ultimately be needed to better understand the biological role of this new proteasome interactor.

## Supporting Information

Figure S1 **Recombinantly produced Bpa sample exhibits the expected molecular weight and is more than 95% pure.** A, Electron spray ionisation mass spectrometry of the purified Bpa sample used in this study. The expected mass for the full length construct after TEV cleavage of the His-tag which leaves behind a Gly residue and accounting for the additional two residues stemming from the chosen expression vector (Leu, Lys) is 19683.2 Da. B, Coomassie stained SDS-gel of 100 pmol purified Bpa or BpaΔHbYX. Asterisk indicates a minor protein contaminant, most likely *E. coli* Hsp70.
(TIF)
