# Supplementary figures and images for: Bacterial Proteasome Activator Bpa (Rv3780) Is a Novel Ring-Shaped Interactor of the Mycobacterial Proteasome
Source: PLoS One. 2014 Dec 3;9(12):e114348. doi: 10.1371/journal.pone.0114348 (PMC4254994; doi:10.1371/journal.pone.0114348)

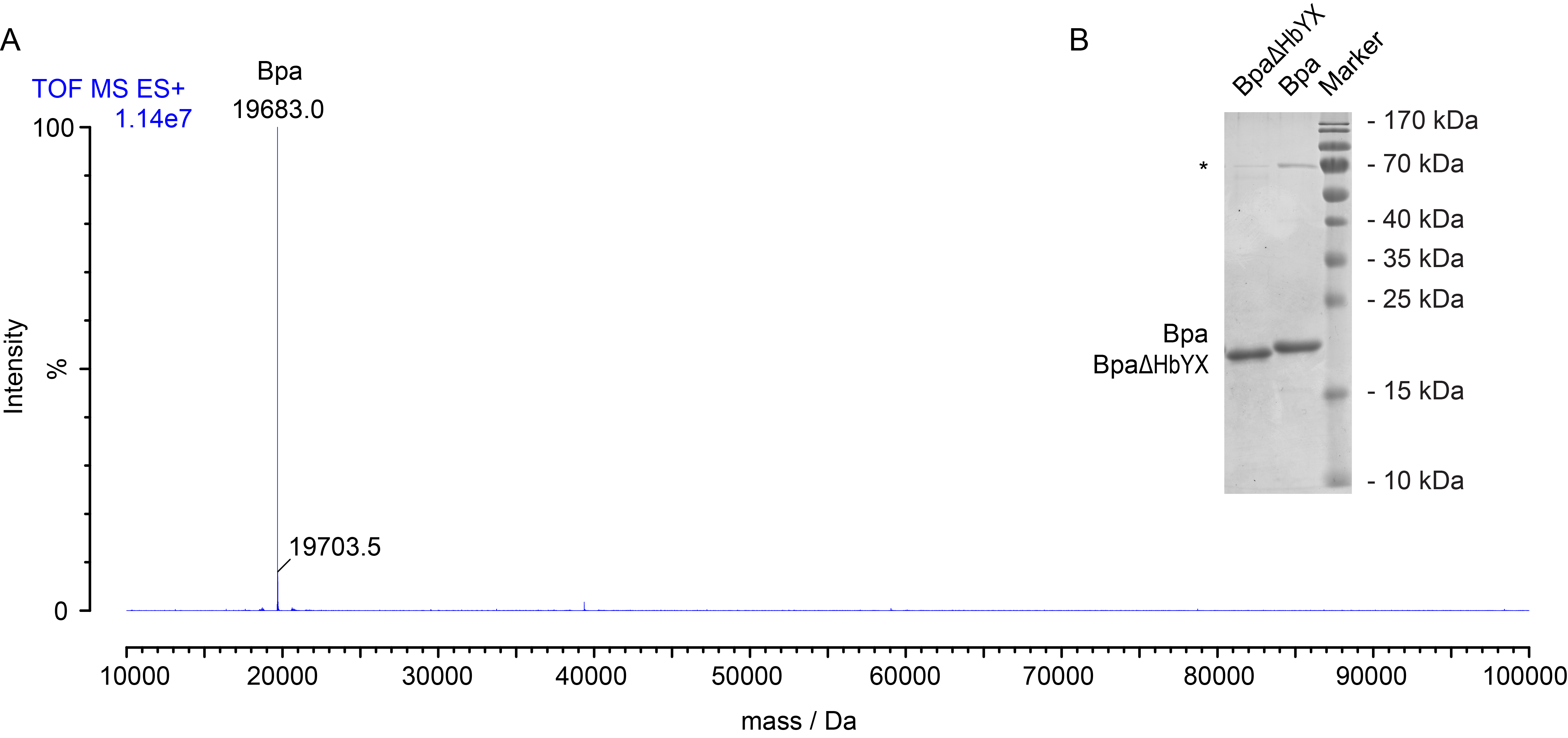

Supplement: Figure S1 — Recombinantly produced Bpa sample exhibits the expected molecular weight and is more than 95% pure. A, Electron spray ionisation mass spectrometry of the purified Bpa sample used in this study. The expected mass for the full length construct after TEV cleavage of the His-tag which leaves behind a Gly residue and accounting for the additional two residues stemming from the chosen expression vector (Leu, Lys) is 19683.2 Da. B, Coomassie stained SDS-gel of 100 pmol purified Bpa or BpaΔHbYX. Asterisk indicates a minor protein contaminant, most likely E. coli Hsp70. (TIF) [file pone.0114348.s001.tif]
